# Severe anemia due to pharyngeal leech infestation; a case report from Ethiopia

**DOI:** 10.1186/s12893-017-0298-7

**Published:** 2017-10-12

**Authors:** Nebiyu Shitaye, Segenet Shibabaw

**Affiliations:** 1 Department of Surgery, Addis Ababa University College of Medicine and Health Science, Tikur Anbesa Specialized Teaching Hospital, Addis Ababa, Ethiopia; 2 Department of public health, Bahir Dar University College of Medicine and Health Science, Felege Hiwot Referral Hospital, Bahir Dar, Ethiopia

**Keywords:** Anemia, Leech, Larynopharynx

## Abstract

**Background:**

Leeches are rare blood-sucking endoparasites. Swimming in streams and ponds as well as drinking contaminated water are the major ways to acquire leeches.

**Case presentation:**

A 6 year old boy who came with a history of hematemesis, frequent spitting of blood stained saliva, fatigue and anorexia to a rural hospital in Ethiopia. This was a rare case of severe anemia caused by a single leech infestation of laryngopharynx that required blood transfusion.

**Conclusion:**

Leech infestation should be considered as a source of unexplained hematemesis, spitting of blood and severe anemia in rural areas.

## Background

Leeches are rare blood-sucking endoparasites and live in contaminated water and can cause potentially fatal complications [1]. Leeches have two suckers namely the anterior and posterior sucker. The anterior sucker consists of jaw and teeth and it is used for suckling of blood. The posterior sucker is primarily used for leverage [2]. Swimming in streams and ponds as well as drinking infested water are the major contamination ways of leech infestation. When the infested water is taken the leech may adhere to the mucosa of upper part of aerodigestive tract. They may present in the nasal cavity, pharynx, larynx, trachea and esophagus. Leeches attach to the mucous membrane and ingest blood [3]. Though leech infestation is uncommon, Patients infested may develop severe anemia because of too much blood loss [4, 5].

In the present report, a 6 year old child with hematemesis and frequent spitting of blood that turned out to be caused by pharyngeal leech infestation is presented.

## Case presentation

A 6 year old child presented to our surgical emergency OPD, chagni primary hospital with hematemesis and frequent spitting of blood stained saliva of 4 days duration. He had also fatigue, headache and anorexia. He has lived in a rural village, Awi zone area using unfiltered water from the spring. He had no history of dysphagia, trauma, drug intake or foreign body ingestion. His family reported that no history of previous systemic illness and he was completely normal 4 days before his presentation.

At presentation the patient was acutely sick looking with paper white pale conjunctiva. He was markedly tachycardic (144beats/min) and febrile (37.8°c), otherwise no lymphadenopathy in all accessible areas, no hepatosplenomegaly and no sign of bruising or petechial hemorrhages.

Upon examining the mouth and throat; the tongue, palate and the oropharyngeal mucosa was blood strained otherwise normal. Only few Investigations were possible because the laboratory and other diagnostic facilities were limited in our hospital. Hemoglobin was 3.1 g/dl, white cell count and platelets were normal. Blood film was negative. The patient was not found to have other underlying illnesses for anemia; his nutritional status was adequate, no evidence of hook worm on stool microscopy and no evidence of bleeding diathesis.

**Fig. 1:**
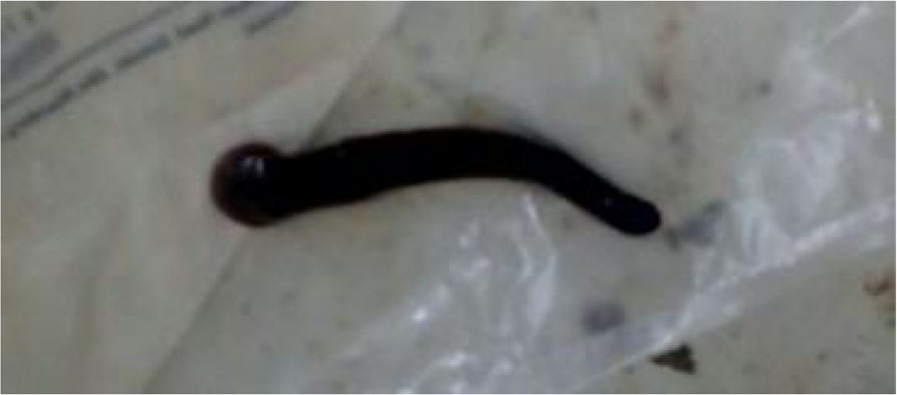
Extracted live leech from the patient which was dark brown in color with a larger posterior and smaller anterior sucker

Blood sample was taken for cross matching as he needs urgent blood transfusion and in the meantime patient was taken to the operation room and examined by direct laryngoscopy. There was a moving brown foreign body in the laryngopharynx. The foreign body was removed gently with the help of foreign body forceps and identified to be a leech measuring 6.2 cm in length (Fig. 1). Bleeding stopped shortly after the leech was removed and patient’s condition significantly improved on subsequent days. He was transfused with 3 units of blood (adjusted for his age and weight) and discharged in a good condition with post transfusion hemoglobin of 7.2 g/dl on his fifth day of admission with haemup syrup supplementation.

## Discussion and conclusions

Leech infestation is described as an important source of hemorrhage and subsequent anemia particularly in children [4–6]. Leeches have ability to feed a volume of blood nearly 10 times their own body weight. This happens after adhering and piercing the host’s mucosa; a process which is painless owing to a local anesthetic released in the leech’s saliva [7]. They get access to the human body either by drinking unfiltered water or through the natural orifices of individuals who swim, play or bath in contaminated water. Owing to this way of spread, nearly all cases have been reported from third world countries where access and use of safe water is limited, particularly in rural settings [2, 6, 9, 10].

Patients’ presentation may differ, depending on the exact site of infestation, but generally signs and symptoms of bleeding may be there, such as hemoptysis, epistaxis, hematemesis, melena, bleeding from the vagina and severe anemia [4, 8]. Salim et al. reported a 12-year old Egyptian boy with frequent dry irritative cough and spitting of blood, endoscopic nasal examination showed nasopharyngeal leech infestations [9]. Kibrab et al. [10] reported 3 cases of vaginal bleeding from Eritrea, in whom speculum examination revealed vaginal leech infestations. In another case, a 15- year -old girl from Tanzania who was presented with a compliant of coughing, chest pain, haemoptysis, pallor, fever and vomiting. Third day after her admission in a hospital, she vomited up a live parasite which confirmed to be a leech [6].

In our presented case of larynopharyngeal leech infestations; hematemesis and frequent spitting of blood stained saliva were the symptoms reported by the patient. Regarding the differential diagnosis variceal bleeding, Mallory-Weiss tears, caustic ingestion, foreign bodies, bleeding disorders, ulcers and gastritis all may give you the clinical picture of the patient.

Considering the geographic setting and age of the pateint variceal bleeding is possible but unlikely in children without signs of portal hypertension. Ulcers and gastritis are more likely in the setting of critical illness or use of nonsteroidal anti-inflammatory drugs (NSAIDs). But this patient was not critically ill and he never used nonsteroidal anti-inflammatory drugs. Mallory-Weiss tears can be considered as a differential diagnosis but patient doesn’t have a history of non-bloody vomiting or retching before the onset of hematemesis. An esophageal foreign body is suggested by a history of a choking episode and drooling of saliva, even if it was transient or occurred days or even weeks before the bleeding episode. There were also no symptoms and signs of bleeding disorder. Even though leech infestation is an uncommon disease, it has to be considered in the differential diagnosis in rural settings.

Leech Removal requires exceptional care and utmost gentleness due to strong attachment with its suckers. The best way to remove the leech from the aerodigestive tract is direct laryngoscopic examination under general anesthesia. For induction sevoflurane-oxygen mixture via facemask is used. The leech is paralyzed and detached from its attachment by effect of sevoflurane. Blunt jaw forceps are preferable for removal. The other important point is removing all parts of the leech’s body. If the head of the leech remains, it might result in continuous blood loss, because the suckers contain and release hirudin. The hirudin is an anticoagulant enzyme. Lidocaine causes relaxation of the head suckers and may be useful [3, 11, 12]. Direct laryngoscopy is gold standard to maintain an emergency diagnosis and removal [11].

In conclusion, the possibility of leech infestation should not be ignored and it has to be considered in the differential diagnosis of unexplained epistaxis, blood spitting and severe anemia especially on those who are living in rural areas where drinking water from springs is a habit. In order to prevent this disease, the communities have to be informed about the importance of using clean and safe water. In addition responsible government bodies should support health education and assist in accessing safe water.

## Abbreviations

BSc: Bachelor of Science
cm: centimeter
Gm/dl: Gram per deciliter
MD: Medical doctor
MPH: Masters in public health
